# Machine‐Learning‐Assisted Discovery and Accelerated Synthesis of Metal Phosphosulfides

**DOI:** 10.1002/smll.74742

**Published:** 2026-08-02

**Authors:** Javier Sanz Rodrigo, Nicholas A. Kryger‐Nelson, Lena A. Mittmann, Eugène Bertin, Ivano E. Castelli, Andrea Crovetto

**Affiliations:** ^1^ National Centre for Nano Fabrication and Characterization (DTU Nanolab) Technical University of Denmark Kongens Lyngby Denmark; ^2^ Department of Energy Conversion and Storage Technical University of Denmark Kongens Lyngby Denmark

**Keywords:** artificial intelligence, band gap, combinatorial synthesis, density functional theory, machine learning algorithm, materials science, phosphorus sulfide

## Abstract

Metal phosphosulfides have emerged as unique multifunctional materials, but they present unique synthesis challenges compared to more established material classes such as oxides and nitrides. As a consequence, experimental development and theoretical understanding of phosphosulfides have focused on individual compounds rather than on accelerated broad‐range exploration. In this work, we first evaluate the synthesizability and band gaps of 909 hypothetical ternary phosphosulfides by density functional theory. We find 19 previously unknown thermodynamically stable compounds, including the first Si‐ and Ge‐based phosphosulfides. For rapid band gap prediction, we then develop a multi‐fidelity machine learning model to translate semilocal density functional theory band gaps into experimentally calibrated band gaps. Importantly, we extend the accelerated material development workflow to the experimental domain by demonstrating a route to high‐throughput synthesis and characterization of virtually any phosphosulfide material system. The method is based on thin‐film combinatorial libraries and yields over 100 unique compositions in each experiment, enabling us to synthesize four distinct phosphosulfide compounds in only four combinatorial experiments without prior synthesis recipes and without compromising on material quality. Thus, we argue that accelerated materials development workflows combining theory, artificial intelligence, synthesis, and characterization can be viable even for experimentally challenging inorganic materials.

## Graphical abstract

The synthesizability and band gaps of 909 hypothetical ternary phosphosulfides are evaluated by a combination of density functional theory and multi‐fidelity machine learning. The accelerated material development workflow is extended to the experimental domain by demonstrating a route to high‐throughput synthesis and characterization of virtually any phosphosulfide material system in thin‐film form.

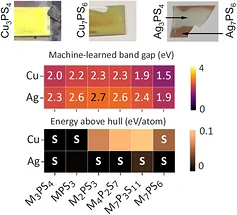

## 1 Introduction

Metal phosphosulfides are an inherently versatile class of materials. As solid solutions between phosphides and sulfides, they allow for gradual property tuning to achieve optimal performance in a variety of applications [1, 2]. As ordered compounds, there are over 200 experimentally reported phosphosulfides with unique properties that can't be simply interpolated from the corresponding sulfides and phosphides [3]. The wide range of phosphorus oxidation states that are accessible in the presence of a more electronegative and a more electropositive species give a plethora of possibilities for materials design.

For example, Li‐based phosphosulfides derived from ${\mathrm{Li}}_{10}$ ${\mathrm{GeP}}_{2}$ $S_{12}$ are among the fastest ionic conductors at room temperature and natural candidates as solid electrolytes in solid‐state batteries [4]. ${\mathrm{CuInP}}_{2}$ $S_{6}$ is an exotic ferroelectric material with record bulk photovoltaic effect performance [5]. Atomically‐thin, antiferromagnetic ${\mathrm{NiPS}}_{3}$ exhibits novel spin‐orbit‐entangled excitons [6]. ${\mathrm{SnP}}_{2}$ $S_{6}$ possesses a unique combination of strong second‐harmonic generation, resistance to laser damage, and phase matchability for a variety of applications in nonlinear optics [7].

Despite their rich property portfolio, development of phosphosulfides has mainly progressed in the form of application‐driven “material silos”, in the sense that discovery of materials with interesting properties has typically been followed by searches for compositionally or structurally similar compounds. Examples are studies on Na‐based phosphosulfides as battery materials following the discovery of Li‐based superionic conductors [8], on van der Waals phosphosulfides following the exfoliation of the first 2D phosphosulfides [9], and on non‐centrosymmetric phosphosulfides as non‐linear optical materials following the discovery of optically active P‐S structural motifs [10]. The materials screening study by Han and Ebert [11], although broader in scope, only considered 18 ternary metal phosphosulfides.

We address two outstanding questions in this paper. First, how do application‐agnostic properties of phosphosulfides (synthesizability and band gap) vary with composition and structure? We provide some answers based on the calculation of thermodynamic stability and band gaps of 909 ternary phosphosulfides through a combination of density functional theory (DFT) and multi‐fidelity machine learning (ML). Along the way, we discover 19 previously unreported ternary phosphosulfides predicted to be thermodynamically stable.

All previously reported synthesis procedures for phosphosulfides known to us are serial, low‐throughput processes. Even in papers that report many different compounds [9, 13, 14], the materials were synthesized by one‐material‐at‐a‐time approaches that lack the ability to screen a range of material compositions and/or process parameters in parallel. Hence, the second question we address is: Can experimental development of phosphosulfides be accelerated by modern high‐throughput methods, despite various challenges in dealing with phosphorus and sulfur in experimental settings? More broadly, this question is of interest for any class of materials where volatile, corrosive, and/or toxic synthesis precursors are necessary. Such a definition arguably includes most inorganic material families that are not oxides, nitrides or intermetallics. By realizing a previously presented vision for a high‐throughput experimental setup compatible with “difficult” chemistries [3], we demonstrate rapid synthesis of four phosphosulfide compounds with high crystalline quality in just a few combinatorial experiments.

## 2 Results

### 2.1 Common Compositions and Anion Structures

**FIGURE 1:**
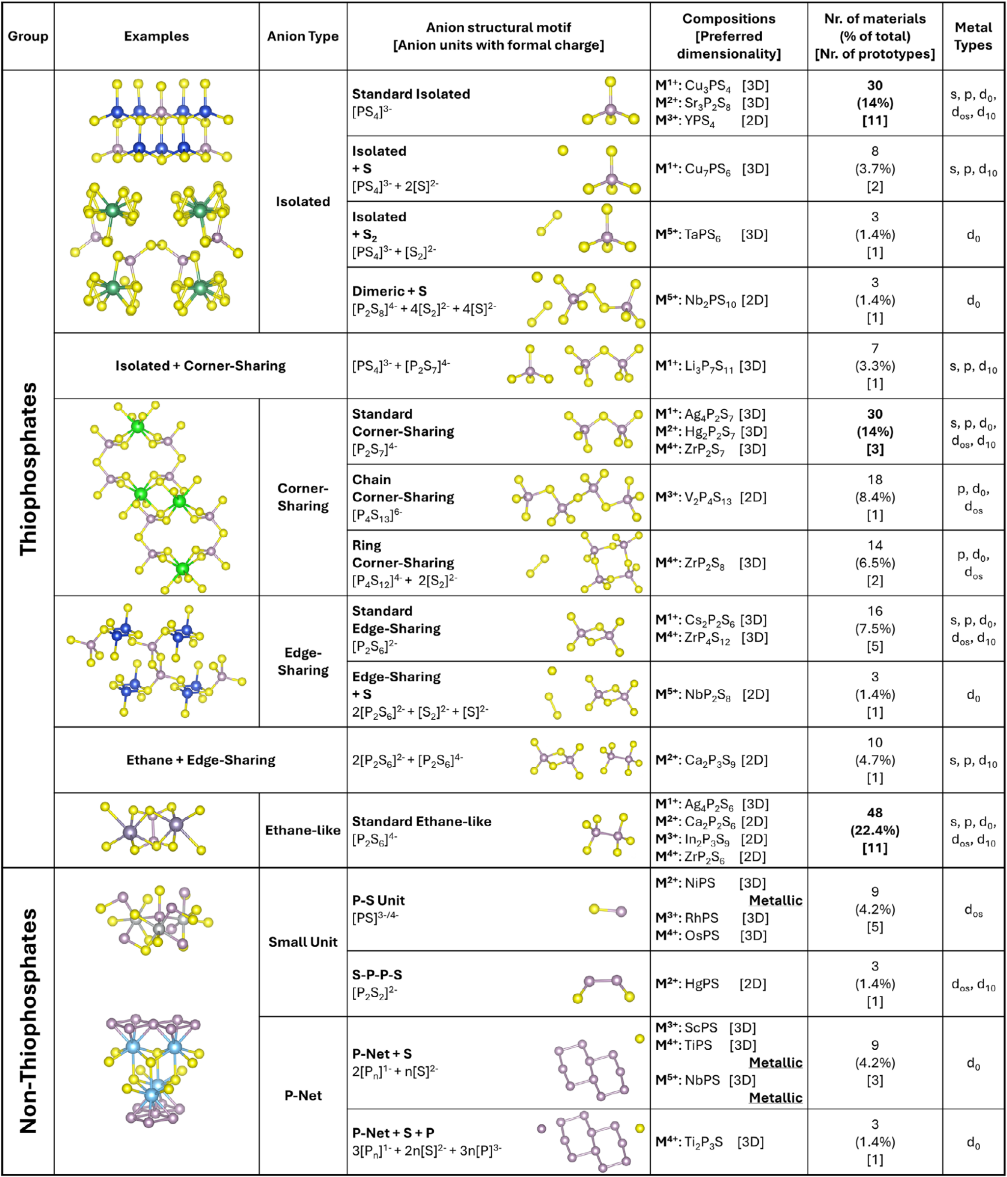
Summary of the structural and compositional diversity of phosphosulfides. The structures are visualized with VESTA [12]. Assignment of oxidation states is discussed in the Supporting Information. The “number of materials” (and the corresponding “% of total”) refers to the number of phosphosulfides calculated in this study that were found to be both lowest‐energy polymorphs and within the 100meV/atom stability tolerance defined in the main text. When these numbers are in bold font, they indicate the three most common structural motifs. A full list of prototypes is given in Table S4. When a composition is labeled as “metallic”, it means that it is not charge balanced. The “metal types” are the blocks of the periodic table containing metals that are compatible with the corresponding structural motif. Among d‐block metals, $d_{0}$, $d_{10}$, and $d_{\mathrm{os}}$ refer to the electronic configuration of the metals after compound formation, with $d_{\mathrm{os}}$ standing for open‐shell configurations.

We classify all the phosphosulfides calculated in this study as either thiophosphates or non‐thiophosphates (Figure 1). In thiophosphates, phosphorus is present in a high positive oxidation state and bonds to sulfur but not to the metal M. This behavior is analogous to phosphates, hence the name. Phosphorus in thiophosphates can be seen either as a metal cation or as part of a polyanionic P‐S motif with an overall negative oxidation state. In this paper we adopt the latter approach and visualize the many different thiophosphate polyanion geometries and formal oxidation states in Figure 1. In non‐thiophosphate compounds, phosphorus takes a negative or neutral oxidation state and bonds to the metal. Phosphorus may also bond to sulfur and to other phosphorus atoms, depending on the specific non‐thiophosphate structure (see Figure 1).

**FIGURE 2:**
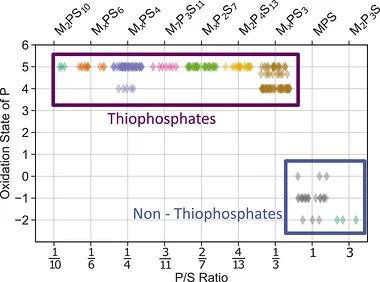
Oxidation state of phosphorus as a function of the atomic P/S ratio in the ternary phosphosulfides calculated in this study (only lowest‐energy polymorphs within stability tolerance). The P/S ratios are discrete and are linked to the generalized compositions shown on the top x axis. The data points are jittered for a more intuitive visualization of the number of materials at each P/S ratio.

Thiophosphates are by far the dominant class of known phosphosulfides, as they make up 85% of the experimentally synthesized ternary phosphosulfides (72 in total). We find that the transition between thiophosphates and non‐thophosphates is well described by the atomic P/S ratio in the material (Figure 2). When $P/S\leq \frac{1}{3}$, phosphorus is in the +5 or +4 oxidation state and the material is a thiophosphate. When $P/S>\frac{1}{3}$, phosphorus switches to oxidation states of ‐2, ‐1, and 0 (Figure 2), marking the transition from thiophosphates to non‐thiophosphates. Note that phosphorus is never present in the ‐3 oxidation state typical of III–V semiconductors, because even when P bonds to M in non‐thiophosphates, it also bonds to either S or other P atoms [15].

Figure 1 summarizes the structural chemistry, elemental compositions, dimensionality, and occurrence of phosphosulfide anions found in at least a few different ternary phosphosulfides close to the stability hull. The structural chemistry of thiophosphates was also partially discussed in previous work [10, 16]. The basic building block of thiophosphate polyanions is the ${\mathrm{PS}}_{4}$ tetrahedron. When this basic unit is not connected to other polyanionic motifs, we refer to it as the “isolated” [${\mathrm{PS}}_{4}$]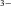 polyanion, one of the most common polyanions encountered in thiophosphate compounds. The isolated ${\mathrm{PS}}_{4}$ tetrahedron can be found together with [$S_{2}$]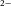 dimers or with individual [S]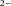 anions or both, giving rise to materials with $M_{x}$ ${\mathrm{PS}}_{6}$ and $M_{2}$ ${\mathrm{PS}}_{10}$ compositions.

In the “corner‐sharing” family of thiophosphates (Figure 1) ${\mathrm{PS}}_{4}$ tetrahedra are connected by sharing corners. The arrangement of corner‐sharing tetrahedra can simply be two units (a ${\left[P_{2}S_{7}\right]}^{4-}$ polyanion, the third most common type in phosphosulfides), a linear chain, or a ring. These three arrangements are present in materials with compositions $M_{x}P_{2}S_{7}$ and $M_{2}P_{4}S_{1}3$, and $M_{4}P_{2}S_{8}$, respectively. Materials with $M_{7}P_{3}S_{1}1$ composition feature both isolated ${\mathrm{PS}}_{4}$ tetrahedra and corner‐sharing ${\mathrm{PS}}_{4}$ tetrahedra pairs in equal amounts.

In the “edge‐sharing” family, a pair of ${\mathrm{PS}}_{4}$ tetrahedra is connected by sharing an edge (a ${\left[P_{2}S_{6}\right]}^{2-}$ polyanion), giving rise to materials with $M_{x}P_{2}S_{6}$ composition. When the edge‐sharing motif is present alongside [$S_{2}$]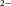 dimers and individual [S]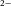 anions, the materials have ${\mathrm{MP}}_{2}S_{8}$ composition.

Interestingly, the “ethane‐like” polyanion is the most common in thiophosphates (Figure 1) despite being the only thiophosphate polyanion that is not based on ${\mathrm{PS}}_{4}$ tetrahedra. Instead, two trigonal pyramidal ${\mathrm{PS}}_{3}$ units are linked by a P‐P bond, similar to the geometry of the ethane molecule. It is compatible with metals with all oxidation states up to +4, resulting in materials with $M_{x}P_{2}S_{6}$ compositions. Ethane‐like and edge‐sharing polyanions can simultaneously be present in equal amounts in the same materials, giving rise to $M_{2}P_{3}S_{9}$ compositions.

Among non‐thiophosphates, three anionic motifs for the MPS composition are found: the P–S unit, made of a single P–S bond which is bonded to metal atoms from both ends; the S–P–P–S motif, made of two P–S units bonded to each other through a P–P bond; and the phosphorus net + sulfide anion motif, in which the metal bonds to a plane of P atoms and to individual sulfide anions. Interestingly, the S–P–P–S motif with its bonded metal atoms is structurally very similar to the ethane‐like polyanion, with the trigonal pyramidal ${\mathrm{PS}}_{3}$ units of the ethane‐like polyanion replaced by trigonal pyramidal ${\mathrm{PM}}_{2}S$ units.

The only experimentally known phosphosulfide composition with P/S>1 ($M_{2}P_{3}S$, generalizable to $M_{2}$ $P_{3-x}$ $S_{1+x}$ with partial anion disorder) is found for Group 4 metals [17]. Its anion motif consists of phosphorus nets, sulfide anions, and phosphide anions. The two motifs with phosphorus nets are also the only ones where P–S bonds are absent, an otherwise ubiquitous feature in known phosphosulfides.

All known thiophosphates are charge‐balanced compounds with well‐defined polyanion oxidation states. Conversely, the MPS composition includes examples of thermodynamically stable, previously synthesized compounds that are intrinsically charge imbalanced and therefore metallic, e.g., NiPS, TiPS, and NbPS. Furthermore, the MPS composition allows for charge‐neutral, semiconducting compounds from metals with various oxidation states (+2, +3, and +4).

The above discussion indicates that the P/S ratio alone does not uniquely describe the structure of the polyanions. For example, the ratio $P/S=\frac{1}{4}$ is present in three thiophosphate classes with very different motifs: Isolated [${\mathrm{PS}}_{4}$]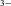 polyanions (${\mathrm{Cu}}_{3}{\mathrm{PS}}_{4}$); rings of corner‐sharing ${\mathrm{PS}}_{4}$ tetrahedra (${\mathrm{ZrP}}_{2}S_{8}$); and edge‐sharing ${\mathrm{PS}}_{4}$ tetrahedra plus [$S_{2}$]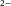 dimers and individual [S]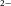 anions (${\mathrm{NbP}}_{2}S_{8}$).

Almost all known structural prototypes for ternary phosphosulfides consist of three‐ or two‐dimensionally bonded structures [3]. When considering the most stable polymorph at each composition, 76% of phosphosulfides located on the stability hull are 3D. For many applications where high‐mobility electronic transport in all directions is desirable, 3D dimensionality is an advantage [3, 18].

### 2.2 Thermodynamic Stability and Synthesizability

Two factors can contribute to positive non‐zero values of energy above the stability hull $E_{h}$ for a material $M_{x}P_{y}S_{z}$: (1) Another polymorph with $M_{x}P_{y}S_{z}$ composition but a different structure has a lower energy; or (2) the energy of $M_{x}P_{y}S_{z}$ lies above the plane connecting three stable compounds in the M‐P‐S convex hull, and therefore decomposes into these compounds. Throughout this study, we only consider the lowest‐energy polymorph at each composition in our analysis, unless otherwise specified. Furthermore, we define a “stability tolerance” of $0\leq E_{h}\leq 100\;\mathrm{meV}/\mathrm{atom}$, which we apply to exclude significantly unstable materials from most of our analysis, unless otherwise specified.

**FIGURE 3:**
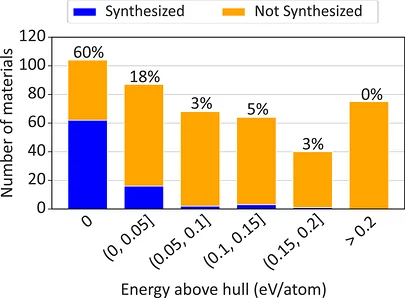
Number of synthesized phosphosulfides as a function of their energy above the stability hull. Only the lowest‐energy polymorphs found in study are included in the statistics. The percentage of synthesized materials for each $E_{h}$ range is indicated.

60% of the screened phosphosulfides that lie on the stability hull have been synthesized (Figure 3). The percentage of synthesized phosphosulfides rapidly drops for materials above the hull (metastable), with a synthesis rate of only 3% when $E_{h}$ is between 50 and 100meV/atom, and of 2% when it is above 100meV/atom. Thus, the energy above hull metric appears to be a good descriptor for the synthesizability of phosphosulfides. Even moderate values of $E_{h}$ generally seem to pose a significant barrier to synthesis. In fact, sulfides and phosphides have previously been shown to be some of the material families with the lowest median $E_{h}$ among synthesized compounds, in contrast to e.g. nitrides, where half of the metastable compounds that have been synthesized have $E_{h}>100\;\mathrm{meV}/\mathrm{atom}$ [19]. We assume that the main sources of error in the calculated $E_{h}$ are the neglect of entropy [20], energy‐lowering disorder [21], and inaccuracies in the elemental reference energies [22].

**FIGURE 4:**
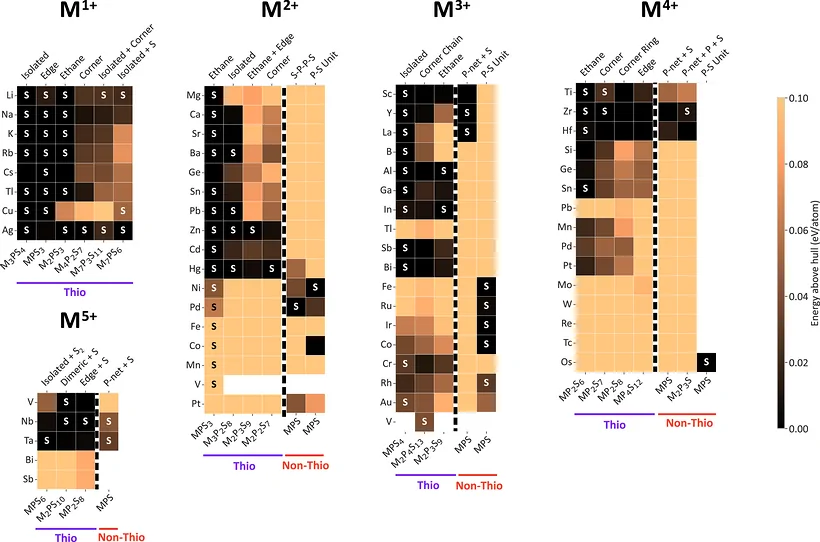
Energy above hull heat maps of all the lowest‐energy phosphosulfide polymorphs calculated in this study. Each $M^{x+}$ heat map includes phosphosulfides of metals with assumed oxidation state x. Along the x axes, materials with different anion motifs (top) are presented, which correspond to specific compositions (bottom). The motifs are visualized in Figure 1. The color of the materials with $E_{h}=100\;\mathrm{meV}/\mathrm{atom}$ is also used for materials with higher energies above hull. White cells indicate that the corresponding material has not been calculated. Cells marked with an “s” indicate that the material is present in the ICSD database and is therefore identified as synthesized. The dashed vertical lines separate thiophosphate compositions (”Thio”) from non‐thiophosphate compositions (”Non‐Thio”). The lowest‐energy prototypes of each material are shown in Figure S1.

Through our screening procedure, 19 new ternary phosphosulfide compositions with a high synthesizability likelihood ($E_{h}=0$) have emerged. They correspond to the black cells in Figure 4 that are not marked with an “s”. These unexplored materials are listed in Table S5, and include four Hf‐based, three Zr‐based, two Sc‐based, and two Cs‐based phosphosulfides. Furthermore, we find thermodynamically stable phosphosulfides (${\mathrm{SiP}}_{2}S_{6}$ and ${\mathrm{GePS}}_{3}$) in the Si–P–S and Ge–P–S systems, in which no ternary compound had been known until now. We also find 71 more new metastable phosphosulfide compositions with moderate energies above the hull (<50meV/atom), which may also be synthesizable but will probably present more challenges. The remaining new compositions (241) lie more than 50meV/atom above the stability hull, and most of them will probably be very challenging to synthesize. Interestingly, the synthesized phosphosulfides beyond the 100meV/atom metastability threshold all belong to the same class of ${\mathrm{MPS}}_{3}$ thiophosphates based on the ethane‐like polyanion coupled with open shell transition metals Fe, Co, Mn, and V [23]. This behavior may be due to the limited number of magnetic configurations [24] considered in our high‐throughput study (see Supporting Information for details).

Heavy p‐block metals often retain their two s‐shell valence electrons (inert pair effect) are therefore partially oxidized in such cases. The preferred oxidation state of these metals in phosphosulfide materials can be inferred from Figure 4 by inspecting energies above hull in their two possible oxidation states. The result is that Sn and Ge can appear both in the +2 and +4 oxidation states, whereas ${\mathrm{Tl}}^{+}$, ${\mathrm{Pb}}^{2+}$, ${\mathrm{Sb}}^{3+}$, and ${\mathrm{Bi}}^{3+}$ are highly favored over ${\mathrm{Tl}}^{3+}$, ${\mathrm{Pb}}^{4+}$, ${\mathrm{Sb}}^{5+}$, and ${\mathrm{Bi}}^{5+}$.

Some compositions result in stable, synthesized materials with almost all metals with a compatible oxidation state. Such compositions are $M_{3}{\mathrm{PS}}_{4}$, ${\mathrm{MPS}}_{3}$, and $M_{2}{\mathrm{PS}}_{3}$ with monovalent metals, ${\mathrm{MPS}}_{3}$ with divalent metals, and ${\mathrm{MPS}}_{4}$ with trivalent p‐block and $d_{0}$ metals (Figure 4). The known non‐thiophosphate structural prototypes seem to only be compatible with transition metals. Structures with P–S and S–P–P–S motifs (MPS composition) are only adopted by open‐shell transition metal cations. Structures with phosphorus nets ($M_{2}P_{3}S$ and MPS compositions) are only adopted by $d_{0}$ cations. On the other hand, thiophosphates of open‐shell transition metals based on the known structural prototypes are never on the stability hull (Figure 4). We note that certain compositions have a very small number of known prototypes (Figure 1). Especially for these compositions, the true lowest‐energy polymorph may be absent from our list of our tested prototypes. Therefore, the values of $E_{h}$ given for these material classes should generally be considered as upper limits.

### 2.3 Thin‐Film Synthesis of ${\mathrm{Cu}}_{3}{\mathrm{PS}}_{4}$, ${\mathrm{Ag}}_{3}{\mathrm{PS}}_{4}$, ${\mathrm{Cu}}_{7}{\mathrm{PS}}_{6}$, and ${\mathrm{Ag}}_{7}{\mathrm{PS}}_{6}$

**FIGURE 5:**
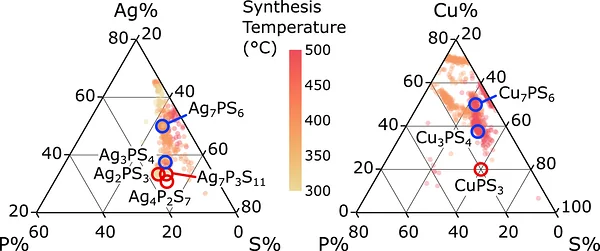
Ag‐P‐S and Cu‐P‐S ternary systems explored by high‐throughput experiments. The data points correspond to the compositions obtained from combinatorial synthesis. The three axes indicate the atomic composition of each of the three elements, normalized to a sum of 100%. Depending on synthesis conditions such as temperature, these data points may be single‐phase compounds (e.g., ${\mathrm{Ag}}_{3}{\mathrm{PS}}_{4}$) like the computationally screened ones, but also multi‐phase systems (e.g., a mix of ${\mathrm{Ag}}_{2}S$ and ${\mathrm{Ag}}_{7}{\mathrm{PS}}_{6}$) or solid solutions (e.g. ${\mathrm{Ag}}_{2+3x}\left(P_{x}S\right)$). Single‐phase compounds synthesized in this study are marked as blue circles. Other compounds calculated in this study are marked as red circles.

The ternary phosphosulfides reported in the ICSD database and marked as “synthesized” in Figure 4 have been grown as bulk crystals and powders, with some examples of nanoparticle synthesis, [26] and very thin, small flakes of phosphosulfides with 2D dimensionality [9]. Many applications in optics, electronics, and energy rely on large‐area growth and crystallization of materials as thin films in the 0.1–1 μm thickness range. As previously discussed, [3] thin films of any phosphosulfide compound are still virtually unreported. In Figure 5, we demonstrate rapid experimental exploration of two selected ternary systems (Ag–P–S and Cu–P–S) in thin‐film form using the recently proposed vacuum‐based DADMARS growth technique. This high‐throughput combinatorial synthesis method, described in detail elsewhere [27], was developed to accelerate materials discovery in experimentally challenging material systems. Each combinatorial synthesis run gives us access to about 100 unique compositions, for which the fraction of each element, crystal structure, and band gaps can be rapidly characterized using automated mapping stages.

We were able to synthesize four single‐phase thin‐film compounds (${\mathrm{Cu}}_{3}{\mathrm{PS}}_{4}$, ${\mathrm{Cu}}_{7}{\mathrm{PS}}_{6}$, ${\mathrm{Ag}}_{3}{\mathrm{PS}}_{4}$ and ${\mathrm{Ag}}_{7}{\mathrm{PS}}_{6}$) with only four combinatorial experiments and without pre‐existing knowledge of synthesis recipes. Remarkably, two single‐phase compounds (${\mathrm{Ag}}_{3}{\mathrm{PS}}_{4}$ and ${\mathrm{Ag}}_{7}{\mathrm{PS}}_{6}$) were obtained in a single combinatorial synthesis run. We define a synthesis success rate metric as the number of synthesized single‐phase materials divided by the number of materials with $E_{h}\leq 50\;\mathrm{meV}/\mathrm{atom}$ that we experimentally covered in composition space (see Figure 5). According to this metric, the overall synthesis success rate was 75%.

**FIGURE 6:**
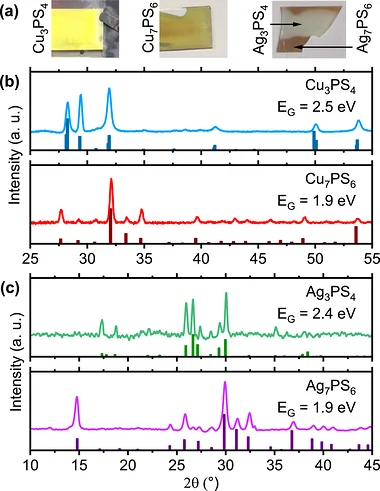
Demonstration of thin‐film synthesis of four phosphosulfides calculated in this work. (a) Photographs of the films grown on glass substrates. Note that the two Ag–P–S compounds were synthesized on the same substrate in the same combinatorial synthesis run. (b,c) Experimental XRD patterns of the four synthesized materials (continuous lines). The vertical bars are the expected positions of the XRD reflections for the lowest‐energy polymorphs calculated in this study, except for ${\mathrm{Cu}}_{7}{\mathrm{PS}}_{6}$ (second‐lowest energy polymorph). ICSD IDs for these reference polymorphs are listed in Table S8. The relative heights of the bars correspond to the predicted peak intensities for a randomly oriented material, which is often not the case in thin films [25]. The experimental band gaps obtained in this work are indicated.

The visual appearance and XRD patterns of the films are shown in Fig. 6. ${\mathrm{Cu}}_{3}{\mathrm{PS}}_{4}$ and ${\mathrm{Ag}}_{3}{\mathrm{PS}}_{4}$ feature the isolated ${\mathrm{PS}}_{4}$ tetrahedron motif, whereas ${\mathrm{Cu}}_{7}{\mathrm{PS}}_{6}$ and ${\mathrm{Ag}}_{7}{\mathrm{PS}}_{6}$ feature isolated ${\mathrm{PS}}_{4}$ tetrahedra combined with $S^{2-}$ anions. These four materials are predicted to be on the stability hull, except for ${\mathrm{Cu}}_{7}{\mathrm{PS}}_{6}$, which lies 51meV/atom above (Figure 4) but may be disorder‐stabilized similar to ${\mathrm{Cu}}_{7}{\mathrm{PSe}}_{6}$ [28]. This hypothesis is supported by the fact that ${\mathrm{Cu}}_{7}{\mathrm{PS}}_{6}$ is the only material that experimentally crystallized in its second‐lowest energy polymorph rather than in its predicted ground state (9meV/atom energy difference).

The synthesized films are air‐stable semiconductors with band gaps in the visible. Visually darker films correspond to lower band gaps (Figure 6a). Standard band gap extraction methods based on optical transmission and reflection measurements (Figure S6) yield the experimental band gaps shown in Table 1, with an estimated uncertainty of ±0.1eV (sources of error are discussed in the Supporting Information). For comparison, we also show in‐house calculated band gaps by four methods, two based on DFT calculations (PBEsol and HSE functionals), and two based on data‐driven corrections to the PBEsol gaps. The two correction methods are: (1) Polynomial fitting between PBEsol band gaps and HSE band gaps; (2) Machine learning (ML) of band gaps from PBEsol fidelity to experimental fidelity. As expected, the PBEsol level suitable for high‐throughput calculations highly underestimates the band gaps of these compounds, whereas the more computationally demanding HSE band gaps are in very good agreement with experiment (Table 1). Among the two methods to improve the quality of PBEsol band gaps, the multifidelity ML approach performs better than the simple PBEsol‐to‐HSE fitting approach, with a mean absolute prediction error (MAE) of 0.24eV with respect to experiment. This error is expected to improve with a larger statistical sample of materials, since the MAE of the same model on a much larger test dataset was 0.17eV (see Methods section). Thus, rapid calculation of PBEsol band gaps followed by their ML‐based extrapolation to experimental fidelity can be a useful for materials screening, where higher‐accuracy first‐principles methods would be too computationally demanding.

**TABLE 1:** Band gaps of the four synthesized compounds using different methods. The experimental band gap of ${\mathrm{Ag}}_{3}{\mathrm{PS}}_{4}$ is marked with an asterisk because the optically determined band gap is 2.7eV, but optical transitions in ${\mathrm{Ag}}_{3}{\mathrm{PS}}_{4}$ were found to be dipole‐forbidden up to 0.3eV above its fundamental band gap [11]. HSE band structures are shown in Figure S8. The mean absolute error (MAE) of each method with respect to our in‐house experimental band gaps is indicated in the bottom row.

	Band gap (eV)				
Material	Experiment	PBEsol	HSE	Fit to	Machine
	(thin film)			HSE	learned
${\mathrm{Cu}}_{3}{\mathrm{PS}}_{4}$	2.5	1.08	2.53	1.96	2.04
${\mathrm{Cu}}_{7}{\mathrm{PS}}_{6}$	1.9	0.70	1.68	1.41	1.52
${\mathrm{Ag}}_{3}{\mathrm{PS}}_{4}$	2.4*	1.04	2.23	1.91	2.30
${\mathrm{Ag}}_{7}{\mathrm{PS}}_{6}$	1.9	0.94	2.05	1.77	1.91
**MAE**		**1.24**	**0.14**	**0.41**	**0.24**

### 2.4 Band Gaps

**FIGURE 7:**
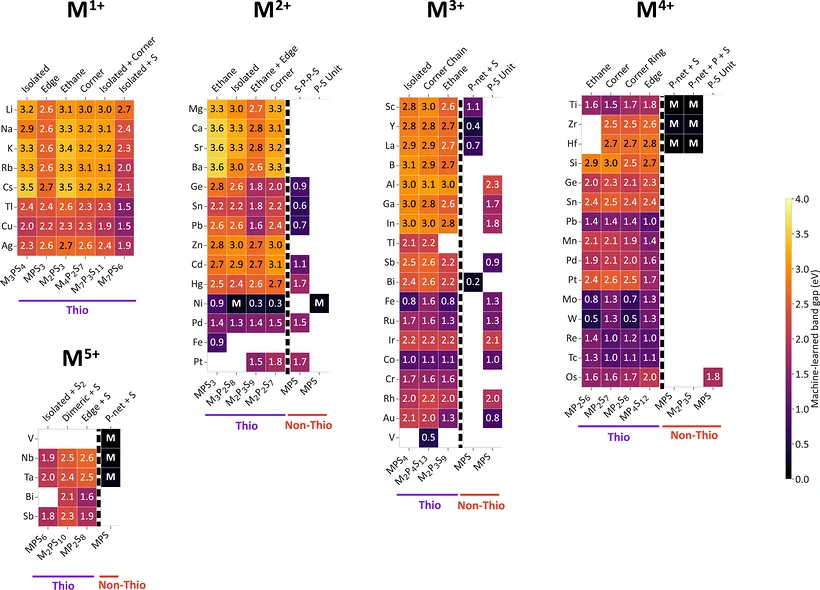
Band gap heat maps of lowest‐energy phosphosulfide polymorphs calculated in this study. Both the color and the number in each cell indicate the band gap predicted by our multi‐fidelity ML model taking elemental composition and PBEsol band gap as inputs, with a mean absolute error of 0.14eV with respect to experimental band gaps in the full test set used when building the model (Table S7). When a cell is marked as “M”, it means that the model predicts that the compound is a metal or has a ML band gap below 0.2eV. White cells indicate that a band structure for the corresponding material was not calculated. The visualization concept is otherwise analogous to Figure 4.

With a more detailed awareness of the quality of multifidelity ML band gap model, we translate the PBEsol band gaps of our calculated phosphosulfides to the experimental fidelity level (Figure 7). Band gaps span a wide range from metallic to around 4 eV. Overall, the widest band gaps are found in alkali and alkaline earth metals, while open‐shell transition metals often have narrow band gaps. Moving horizontally in each of the heat maps in Figure 7, there are some band gap changes across compositions and polyanion motifs at fixed metal cation. However, the three most common motifs (isolated ${\mathrm{PS}}_{4}$, corner‐sharing ${\mathrm{PS}}_{4}$, and ethane) tend to give similar band gaps for the same M due to their relatively similar bonding geometries. Overall, materials with wider band gaps tend to lie closer to the stability hull, but the correlation is weak.

**FIGURE 8:**
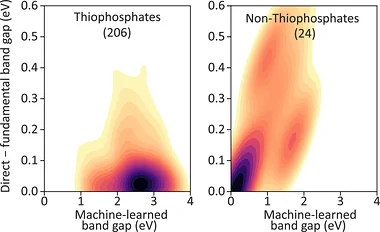
Contour plots showing the (fundamental) band gap predicted by our multi‐fidelity ML model versus the difference between the lowest direct band gap and the fundamental gap, as derived from PBEsol band structures. This difference is greater than zero if the fundamental gap is indirect. Darker colors indicate a higher density of materials. The plot on the left only includes thiophosphates. The plot on the right only includes non‐thiophosphates, as defined in Figure 1. Only lowest‐energy polymorphs within the stability tolerance are included. The number of material entries used in these plots is shown in parentheses.

There are important qualitative differences between thiophosphates and non‐thiophosphates. Thiophosphates occur most frequently in the 2.3–3.0 eV band gap range (Figure 8) with a preference for slightly indirect gaps. Interestingly, the maximum in our ML‐predicted band gap distribution matches the corresponding maximum determined for all known sulfide compounds [29], giving further credibility to our proposed method for accelerated band gap prediction. The median difference between the lowest direct gap and the fundamental gap in thiophosphates is 23meV when only considering indirect gap materials, and 14meV when including both direct and indirect gap materials. Thus, even in indirect gap thiophosphates, the direct gap is typically within thermal energy of the fundamental gap at room temperature. While indirect gaps have historically been considered a drawback for optoelectronic applications, an indirect gap just below a direct one is generally benign or even beneficial for optoelectronic energy conversion when the device is limited by charge collection [30, 31]. Thiophosphates are therefore well positioned as potential optoelectronic materials for a variety of applications where band gaps in the yellow‐blue region are desirable, such as light emitting diodes, photocatalysis, and solar fuels. According to Figure 7, thiophosphates seem to have a very low propensity for metallic band structures.

On the other hand, non‐thiophosphates have a more complex band gap distribution with three local maxima (Figure 8) that are characteristic of the three anion motifs identified in Figure 1. The main underlying reason for a more complex band gap distribution is a motif‐dependent participation of P states to the valence band maximum, due to the highly different bonding environment of P atoms in non‐thiophosphates (Figure 1). The first local maximum is characteristic of materials with the P–S unit (see Figure S9) and is located at (indirect) band gaps around 1.2eV, where the direct band gap lies around 0.4 eV above the indirect one. This maximum remains very clearly defined when the number of materials is increased by a factor 4 by also including higher‐energy polymorphs based on the P–S unit (Figure S9).

The second local maximum is found when approaching the metallic limit (0eV). This maximum is characteristic of non‐thiophosphates based on P nets and $S^{2-}$ anions, either with or without separated $P^{3-}$ anions (Figure S9). Here, we should note that the ML model often predicts the opening of very small band gaps (<0.2eV) in phosphosulfides that are metallic at the PBEsol level. This is also observed in some of the MPS compositions that we predicted to be charge‐imbalanced in Table 1 and therefore intrinsically metallic, indicating an unphysical ML prediction. Since the uncertainty in the ML‐predicted band gap is around 0.2eV (Table 1 and Table S7), we label all materials with predicted band gaps below this threshold as metallic in Figure 7. Distinguishing between true metals and narrow‐gap semiconductors is likely one of the most challenging tasks for the multi‐fidelity ML model and, more generally, for any method trying to determine the band gap of a material where a semilocal DFT functional yields a metallic band structure. Although the ML model is trained on experimental band gaps, experimental methods themselves often struggle to distinguish between metals, semimetals, and semiconductors with very narrow gaps [32].

The third local maximum for non‐thiophosphates is centered around 1.7eV band gap with moderately indirect gaps (on average 0.15eV below the lowest direct gap). This maximum arises from the materials with the S–P–P–S motif. However, this motif only has one known prototype and three lowest‐energy polymorphs within the stability threshold. When higher‐energy materials are included (Figure S9) new maxima appear, indicating that this third maximum is not an intrinsic feature of the S–P–P–S motif.

The combined band gap distributions of thiophosphates and non‐thiophosphates reveal that ternary metal phosphosulfides as a whole cover almost the full spectrum of band gap combinations, from metallic structures to low, moderate, and wide band gaps with various degrees of indirectedness. Only ultra‐wide band gaps above 3.6eV are not represented. Moving to quaternary compounds is expected to further expand possiblities for materials design. The space of non‐thiophosphate compounds features particularly diverse bonding patterns and electronic properties in spite of its small size. Thus, this family appears particularly promising for further discovery of materials with unexpected properties. Many chemically plausible non‐thiophosphates are predicted to be thermodynamically unstable by our calculations (Figure 4). However, these calculations rely on a small range of structural prototypes which reflect the very limited research attention they have received so far, with no experimental reports of any quaternary non‐thiophosphates [3], nor of non‐thiophosphates with metals outside the d block (Figure 4). It remains to be seen whether many synthesizable non‐thiophosphates are still awaiting discovery.

## 3 Conclusions

We evaluated the thermodynamic stability and synthesizability of nearly a thousand ternary phosphosulfides by high‐throughput DFT, discovering 19 previously unknown materials that lie on the stability hull. We found that the synthesizability of phosphosulfides is highly correlated with their energy above hull. Although there are a few examples of synthesized phosphosulfides lying higher than 100meV/atom above the stability hull, synthesis of highly metastable phosphosulfides appears to be more difficult than in the case of oxides and especially nitrides.

We developed a multi‐fidelity machine learning model to rapidly predict experimentally calibrated band gaps with elemental composition and PBEsol band gaps as the sole inputs. By including our in‐house experimental samples in the validation of the multi‐fidelity model, we conclude that the model accuracy is not far from the typical experimental error in measuring the band gap of a synthesized sample without any prior knowledge.

The amphoteric nature of phosphorus, acting either as a cation or as an anion depending on chemical context, results in two very different classes of phosphosulfides: Thiophosphates and non‐thiophosphates. The transition from one class to another occurs at a P/S ratio of 1, coinciding with a switch in the phosphorus oxidation state from positive to negative. Structural motifs, compatible metal cations, band gap distributions, tendency to indirect band gaps, and tendency to metallicity are completely different in thiophosphates and non‐thiophosphates. We expect that these insights can be used to intelligently search for novel phosphosulfides with entirely new compositions, new structures, and higher complexity (quaternary compounds and beyond) in the future.

Importantly, the high throughput of our workflow is not limited to computational aspects but it extends to the experimental domain, including both synthesis and characterization. This feature was enabled by a unique thin‐film synthesis technique (DADMARS) that was specifically designed for accelerated, yet high‐quality synthesis of experimentally challenging material classes [27]. In practice, the high‐throughput experimental setup allowed us to synthesize four phosphosulfides that had not previously been grown as thin films after only four combinatorial synthesis runs. This work demonstrates that accelerated materials development workflows combining theory, AI, and experiment are within reach even for “difficult” chemical systems, as long as a suitable experimental setup has been designed.

## 4 Methods

### 4.1 First‐Principles Calculations

First‐principles calculations were performed using DFT with the projector‐augmented wave method [33] as implemented in the Vienna Ab‐Initio Simulation Package (VASP) [34, 35, 36, 37, 38]. We only considered ternary metal phosphosulfides in structures that had been experimentally reported for at least one phosphosulfide material, according to the Inorganic Crystal Structure Database (ICSD) [39]. In a first screening step, we decorated each known structural prototype with metals with a compatible oxidation state and calculated the relaxed geometry and total energy of each unique compound. The initial geometries were taken from the Material Project database. [40] A full list of the prototypes used in this work, criteria for their selection, and their corresponding Materials Project ID, are available in the Supporting Information (Table S4). All calculations were submitted using the SLURM frontend MyQueue [41].

Based on the preliminary tests shown in the Supporting Information, the PBESol functional was selected for all calculations reported in this paper, unless otherwise specified [42]. The structure relaxation calculations were run on Γ‐centered uniform *k*‐point grids with a 680 eV plane wave energy cutoff and 5 *k*‐points per Å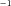 along each reciprocal lattice direction (VASP kspacing parameter: 0.22). The lattice parameters and atomic positions were relaxed with convergence criteria of less than 10meV/Å forces on all atoms, and less than $10^{-6}\;\mathrm{eV}$ total energy difference between steps. All calculations were spin polarized.

The energies above the stability hull were determined by constructing convex hulls for the 50 ternary M–P–S systems considered in this study using pymatgen, as detailed in the Supporting Information. For each ternary compound, $E_{h}$ was calculated with respect to all phases contained in the corresponding convex hull, including elemental phases, binaries, and all calculated ternary phosphosulfides. If $E_{h}=0$, then the compound has a lower energy than its potential decomposition products and is thermodynamically stable. If $E_{h}>0$, the value of $E_{h}$ represents the energy of the compound above the energy of its relevant decomposition products. The structural dimensionality of all the calculated materials was determined using the algorithm by Larsen et al. [43] as implemented in pymatgen [44].

For the materials found to lie within 200meV/atom of the stability hull, we proceeded to a non self‐consistent calculation of their electronic band structure. These calculations were run along high symmetry paths in the Brillouin zone, as defined in Ref. [45], with 20 *k*‐points for each segment in the path and 680 eV plane wave energy cutoff. From the calculated band structures, we extracted the fundamental band gap and lowest direct band gap of each material using pymatgen. For spin‐polarized band structures (about 9% of all band structures within the stability tolerance) we kept the spin channels separate (no spin flipping) and quoted the fundamental and lowest direct band gaps from the spin channel with the lowest fundamental band gaps. For the four materials that we synthesized as thin films, we performed complementary relaxation and band structure calculations using the hybrid HSE06 functional [46, 47] with a plane wave energy cutoff of 500 eV or higher, and 20 *k*‐points for each segment in the path. Further details about the first‐principles DFT calculations are given in the Supporting Information.

### 4.2 Machine/Statistical Learning

We employed two statistical methods to correct PBEsol band gaps. The first method is a simple second‐order polynomial regression between HSE and PBEsol band gaps, using a dataset of 39 ternary phosphosulfides for which HSE band gaps were available on the SNUMAT database [48] and the PBEsol band gaps were calculated in this work. This approach is analogous to the HSE to PBE regression method employed in previous work [3]. The best‐fit parameters and fit quality are shown in Figure S2.

The second method is a multi‐fidelity deep learning approach based on permutation‐invariant transformers. We developed this method to predict the experimental band gaps of inorganic materials from two inputs: (1) chemical composition, and (2) a calculated band gap at one of six possible fidelity levels, corresponding to different DFT functionals (PBE, PBE+U, PBEsol, SCAN, GLLB‐SC, and HSE). For this work, the input fidelity level of interest is PBEsol. The model was partially inspired by Ref. [49], with the important difference that here structural information is not needed.

Two distinct (sub)models were developed to obtain a prediction‐ready multi‐fidelity platform. The base model uses chemical composition as the only input to predict the band gap at a given fidelity level (e.g, PBEsol). The translation model is a two‐input model that converts the band gap between fidelity levels. It uses chemical composition and a known band gap at a given fidelity level (e.g., PBEsol) to predict the band gap at another level (e.g., experimental).

The PBE and PBE+U datasets (91,920 and 21,917 entries respectively) were obtained from the Materials Project database [40]. The PBEsol and SCAN datasets (113,106 and 112,722 entries) were obtained from the database also used to construct our convex hull diagrams [50]. The GLLB‐SC dataset (2,395 entries) was generated in [51] and obtained through MPContribs [52]. The HSE dataset (10,482 entries) was obtained from the SNUMAT database. [48] Finally, the experimental dataset (4,605 entries) was compiled by the authors of a previous ML model [49] based on an earlier compilation [53] and released with the accompanying paper. About half of the materials in this dataset are metals, while the other half have non‐zero band gaps. When preparing the datasets for training, we retained only the band gap of the lowest‐energy polymorph at each composition, as necessary for models not incorporating structural information [54]. Details are available in the Supporting Information.

The mean absolute error (MAE) for the base model to predict experimental band gaps from composition only is 0.37eV (Table S6). The five‐fold cross‐validation MAE for the translation model to predict an experimental band gap from composition and a PBEsol band gap is 0.17eV (Table S7).

### 4.3 Experiments

In line with the rest of this study, the synthesis, experimental characterization, and experimental data management were conducted with high‐throughput methods. Thin‐film combinatorial libraries [55] were synthesized by directional‐and‐diffuse multi‐anion reactive sputtering (DADMARS), a technique presented in depth in a previous publication [27]. Briefly, a metallic (M) sputter source (Cu or Ag), reactive ${\mathrm{PH}}_{3}$ gas, and a cracked sulfur beam were combined in an Ar discharge to deposit M–P–S films with intentional gradients in elemental composition across a large growth area. The synthesis pressure was 0.67Pa, the synthesis temperature was varied between 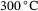 and 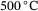, and the fluxes of M, P, and S resulting from these sources were varied from one synthesis run to another to sample different portions of the Cu–P–S and Ag–P–S systems. These fluxes were intentionally inhomogeneous in space to obtain the desired composition gradients allowing for rapid combinatorial exploration. Elemental composition was measured by energy‐dispersive X‐ray spectroscopy (EDX). Structural information was inferred from x‐ray diffraction (XRD). Band gaps were determined from optical transmission and reflection spectra. Further details of the experimental characterization are given in the Supporting Information. High‐throughput experimental data was managed and analyzed with customized workflows in a NOMAD Oasis database [56] with human intervention mainly limited to validation steps.

## Conflicts of Interest

The authors declare no conflicts of interest.

## Supporting information


**Supporting File**:smll74742‐sup‐0001‐SuppMat.pdf

## Data Availability

The computational and experimental data generated from this study, including all DFT calculations and a database of computed properties, is available in the NOMAD database at https://dx.doi.org/10.17172/NOMAD/2026.01.22‐2. The trained multi‐fidelity machine learning model used to translate band gaps is available on https://github.com/nkrygernelson/FETT.
